# Expression Profiling of microRNA From Peripheral Blood of Dairy Cows in Response to *Staphylococcus aureus*-Infected Mastitis

**DOI:** 10.3389/fvets.2021.691196

**Published:** 2021-08-04

**Authors:** Zhuo-Ma Luoreng, Jian Yang, Xing-Ping Wang, Da-Wei Wei, Lin-Sen Zan

**Affiliations:** ^1^Key Laboratory of Ruminant Molecular Cell Breeding of Ningxia Hui Autonomous Region, School of Agriculture, Ningxia University, Yinchuan, China; ^2^College of Animal Science and Technology, Northwest A & F University, Yangling, China

**Keywords:** cow, miRNA, mastitis, *Staphylococcus aureus*, molecular biomarker, RNAseq

## Abstract

As the main pathogen causing dairy cow mastitis, *Staphylococcus aureus* can cause subclinical mastitis, which is difficult to be diagnosed. It seriously affects milk quality and the economic benefits of the dairy industry. Therefore, it is very necessary to find biomarkers for early diagnosis of *S. aureus*-infected mastitis in peripheral blood of dairy cows. In this study, *S. aureus* was used to infect the mammary gland tissues of dairy cows, and a mastitis model was successfully constructed. The RNAseq technology was used to determine the expression profiles of microRNA (miRNA) from peripheral blood of dairy cows infected with *S. aureus* at 0, 1, 3, 5, and 7 days. A total of 288 differentially expressed miRNAs (DIE-miRNAs) were found, of which 108 were known miRNAs and 180 were novel predicted miRNAs. Bioinformatics analysis results showed that the above DIE-miRNAs might be involved in 10 immune system-related signaling pathways (i.e., chemokine signaling pathway, leukocyte transendothelial migration, natural killer cell-mediated cytotoxicity, toll-like receptor signaling pathway, Jak-STAT signaling pathway, MAPK signaling pathway, Wnt signaling pathway, cell adhesion molecules, cytokine-cytokine receptor interaction, and ECM-receptor interaction), thus regulating the process of *S. aureus* mastitis. It was also found that the expression variation of up-regulated expression of miR-320a, miR-19a, and miR-19b as well as down-regulated expression of miR-143, miR-205, and miR-24 reached a significant level on the 5th and 7th day of infection, suggesting that they might play an important biological role in mastitis and provide a direction for the research and development of molecular therapy technology for mastitis. However, at different times after *S. aureus* infection, miR-1301 was significantly up-regulated in peripheral blood. miR-2284r was significantly down-regulated, suggesting that these two miRNAs might be the new blood biomarkers for *S. aureus*-infected dairy cow mastitis. The above results laid a new foundation for the research and development of molecular diagnosis and biological therapy technology for *S. aureus*-infected mastitis in dairy cow.

## Introduction

Dairy cow mastitis is an inflammatory disease caused by the infection of the mammary gland by pathogenic microorganisms, which can decrease milk production and milk quality and increase the mortality and cull rate of dairy cows, thereby affecting the economic benefits of dairy farms. There are more than 150 pathogenic bacteria that cause mastitis, in which, *Staphylococcus aureus* is one of the important contact infectious pathogens, which can cause chronic subclinical dairy cow mastitis (1). About 70–90% of dairy cows with *S. aureus*-infected mastitis are resistant to antibiotics, and about 25% of cows have to stop lactation or milk production due to mastitis (2). Therefore, it is important to explore the molecular immune mechanism of dairy cows against *S. aureus* infection and screen biological molecules for early and rapid diagnosis.

MicroRNAs (miRNAs) belong to a class of highly conserved endogenous non-coding small RNA (sRNA) molecules (3). Mature miRNAs can degrade target mRNA or inhibit translation mediated by target mRNA through binding to the 3′ untranslated region (3′UTR) or 5′ untranslated region (5′UTR) sequence of the target mRNA (4). At present, the regulation of miRNA has become an important regulation method in the mammalian immune system. Although a single miRNA can regulate the protein synthesis of hundreds of target genes, they affect their physiological processes by regulating the concentration of a few key proteins in the cell signal transduction pathway (5). Cow miRNA could control activities of 60% of protein-coding genes and regulate almost every cellular process of mammals (6). In addition, cells related to the immune system express more than 100 miRNAs, affecting the molecular pathways that control innate and adaptive immune responses (7).

In recent years, miRNA expression analysis was done in the mammary gland of experimental mastitis cows (8, 9) and the peripheral blood of clinical mastitis cows (10). miRNAs related to mastitis in dairy cows are affected by factors, such as pathogenic bacteria type, infection dose and infection time, and its expression level changes very sensitively (11). *S. aureus* is the main pathogen of mastitis, but there are a few studies on the dynamic changes of miRNA expression in peripheral blood during the onset and progression of dairy cows with *S. aureus*-infected mastitis. Understanding the changes in miRNA during the pathogenesis is of great significance for the molecular diagnosis and biological therapy of mastitis. Therefore, in this study, a low-dose of *S.aureus* was used to infect the mammary gland tissues of dairy cows to prepare a *S. aureus*-infected mastitis cow model. The studies of the dynamic expression levels of peripheral blood miRNAs at different stages of *S.aureus*-infected mastitis and exploration of biomarkers of *S.aureus*-infected dairy cow mastitis could provide a basis for molecular diagnosis and biological therapy.

## Materials and Methods

### Animals

The experimental animals in this study were lactating 2–3-year-old half-sib Chinese Holstein cows (*n* = 3), which were obtained from a farm in Yangling, Shaanxi, China. They had the same feeding and management environment. The body conditions of the dairy cows were measured 3 weeks before the experiment. There was no abnormality in the feeding, drinking, heartbeat, respiratory rate, and other physiological activities of the experimental cows. Veterinary clinical diagnosis showed that the cows had no any symptoms of disease including mastitis. Furthermore, and milk somatic cell count (SCC) detected by Fast-D™ Mastitis Detector (Zhangjiagang chuangpu Machinery Co., Ltd, China) was <200,000/mL. In addition, three healthy cows (SCC < 200,000/mL) and three clinical mastitis cows (SCC > 500,000/mL) in the same pasture were selected for validation experiment of miRNA biomarkers.

### Construction of Cow Mastitis Model and Peripheral Blood Collection

*S. aureus* (ATCC 25923) were purchased from Shanghai Fuxiang Biotechnology Co., Ltd., China, and the 10^5^ CFU/mL *S. aureus* suspension (9) was prepared according to the manufacturer's instructions. The udders and mammilla of the experimental dairy cows were cleaned and sterilized with 75% ethanol, and 5 mL of 10^5^ CFU/mL *S. aureus* suspension was injected into the udders of the experimental dairy cows through the mammary duct with a milk-passing needle. The body temperature and milk SCC of all cows at different times were measured. On the 7th day of induction, the mammary gland tissues were collected using painless surgery for hematoxylin-eosin (HE) staining and pathological analysis. On the day 0, 1, 3, 5, and 7 after *S. aureus* infection, peripheral blood was collected into tube without silica treatment from the jugular vein, and blood samples of different cows with the same induction time were mixed and stored using RNALater™ RNA stabilization reagent for blood (Beyotime Biotechnology, Tianjin, China). In addition, we also collected the peripheral blood of six *S. aureus*-type clinical mastitis cows. All samples were frozen and stored at −80°C for further analysis.

### RNA Extraction and sRNA Library Construction

TRIzoL kit (Invitrogen, Carlsbad, CA, USA) was used to extract total RNA from all blood samples according to the manufacturer's instructions. RNA of clinical samples were used for qPCR validation of biomarker miRNA. For the RNA of the blood at different induction times (0, 1, 3, 5, and 7 days), Nano Photometer Spectrophotometer (IMPLEN, CA, USA) was used for the RNA purity test. Qubit 2.0 Fluorometer (Life Technologies, CA, USA) and the Agilent Bioanalyzer 2100 system (Agilent Technologies, CA, USA) were used for integrity test to ensure the RNA was intact. A total of 1.5 μg of qualified RNA was taken, and EBNext®Ultra™ sRNA sample pre kit was used to construct the miRNA library according to the product instructions.

### High-Throughput Sequencing and Quality Control

The constructed library was diluted to 1 ng/μL. The Agilent Bioanalyzer 2100 system (Agilent Technologies, CA, USA) was used to detect the size of inserted fragments, and quantitative polymerase chain reaction (qPCR) was used to detect the effective concentration of the library. Sequencing was performed on the Illumina HiSeq2500 high-throughput sequencing platform. Screening and quality control were performed to obtain clean reads according to the methods in accordance with a previous report (11).

### sRNA Classification Annotation

Bowtie software (12) was used to compare clean reads with 4 databases (Silva, GtRNAdb, Rfam and Repbase) for filtering out repetitive sequences and non-coding RNAs, such as ribosomal RNA (rRNA), transfer RNA (tRNA), small nuclear (snRNA), and small nucleolar RNA (snoRNA). Unannotated reads containing miRNA were obtained. miRDeep2 software (13) was used for sequence comparison of unannotated reads, and bovine reference genome was used to obtain mapped reads.

### miRNA Identification

In order to determine the miRNA sequence in unannotated reads, miRDeep2 software (13) was used to compare unannotated reads with genome sequence to obtain possible miRNA precursor sequences. RNAfold and Ranfold were used to calculate precursor structure energy and predict secondary structure. Combined with the information on the miRNA precursor sequence, the Bayesian model was used to score of miRNA Identification. The miRNA with a score >80% was considered to be reliable, and it was compared with the miBase database to determine known miRNA and new miRNA.

### miRNA Expression and Differential Expression Analysis

The read count of miRNA in each sample was counted and normalized according to the following equation, and the tags per million reads (TPM) value of miRNA expression (9, 14) was calculated as follows:

$$\begin{array}{l} \text{TPM }=\;(\text{specific miRNA reads}\times 1,000,000)/\text{total miRNA}\\ \text{ mapped reads} \end{array}$$

IDEG6 software (15) was used to compare and analyze the differential expression of miRNA between control group and induced-group. In order to eliminate false positives, Benjamini Hochberg's correction method was used to correct the *p*-value to the q value in the analysis process (16). Finally, |${\text{log}}_{2}^{\left(\text{FoldChange}\right)}$|≥1, false discovery rate (FDR) ≤ 0.01 and *q* ≤ 0.005 were used as criteria to screen differentially expressed miRNA (DIE-miRNA).

### qPCR Verification of miRNA Expression

A total of 10 DIE-miRNAs (5 up-regulated and 5 down-regulated miRNAs) were randomly selected, and the reverse transcription (RT) and qPCR primers for the stem-loop structure were designed (Supplementary Table 1). Using the total RNA from the peripheral blood of dairy cows at different induction times (0, 1, 3, 5, and 7 days) as a template, PrimeScript™ RT reagent kit with gDNA eraser kit (Perfect Real Time) (Takara Biomedical Technology Co., Ltd., Beijing, China) and TB Green® Premix Ex Taq™ II (Tli RNaseH Plus) kit (Takara Biomedical Technology Co., Ltd., Beijing, China) were used to perform RT and qPCR experiments according to the product instructions, respectively. In shorts, the gDNA Eraser reaction mixture (10 μL total volume contained 2.0 μL of 5 × gDNA Eraser Buffer, 1.0 μL of DNA Eraser, 7.0 μL of RNase Free H_2_O) was mixed with the 1.0 μg total RNA extracted from the peripheral blood. The mixture was incubated at 42°C for 2 min to promote the degradation of gDNA. Immediately following this step: 10.0 μL Mix (1.0 μL of PrimeScropt RT Enzyme, 1.0 μL of 10 μM stem-loop RT Primer, 4.0 μL of 5 × PrimeScript Buffer, and 4.0 μL of RNase Free H_2_O) was added to the 10 μL of gDNA Eraser reaction mixture mentioned above. Then, the mixture was incubated at 37°C for 15 min, 85°C for 5 sec, then immediately placed on ice for 2 min. The cDNA was diluted 1:10 with DNase/RNase-free water. The qPCR was performed in an CFX96 Touch Real-Time PCR Detection System (BIO-RAD, USA) in a total volume of 20 μL [10 μL of 2 × SYBR Premix Ex TaqII (TliRNaseH Plus), 0.8 μL of PCR Forward Primer (10 μM), 0.8 μL of PCR Reverse Primer (10 μM), 2 μL of cDNA (100 ng/μL), and ddH_2_O to a final volume of 20 μL]. The reaction mixtures were incubated at 95°C for 30 s, followed by 40 cycles of 95°C for 5 s, 60°C for 34 s, and 72°C for 30 s.

Glyceraldehyde 3-phosphate dehydrogenase (GADPH) and ribosomal protein S18 (RPS18) were used as dual internal references, and the relative expression of miRNA (mean ± standard deviation, SD) was calculated using the 2^−Δ*ΔCt*^ method. Student's *t*-test was used to test the significance of the difference, and *p* < 0.05 indicated a significant difference. All qPCR experiments and analysis methods were in accordance with the minimum information for publication of quantitative real-time PCR experiments (MIQE) guidelines (17).

The qPCR results of DIE-miRNA were compared with the results of RNAseq. If the expression trends of the qPCR and RNAseq are consistent, the RNASeq and screening results of DIE-miRNA will be reliable.

### Target Gene Prediction and Kyoto Encyclopedia of Genes and Genomes (KEGG) Function Annotation

According to the bovine mRNA and miRNA sequence information obtained from sequencing, miRanda (18) and RNAhybrid software (19) were used to predict DIE-miRNA target genes. In order to explore the function of DIE-miRNA, KOBAS 2.0 software (20) was used to annotate the KEGG signaling pathway for predicted miRNA target genes.

## Results

### Mastitis Model Identification

The SCC in milk was measured at different times when *S. aureus* induced the mammary glands of dairy cows. The results showed that the average values of SCC in milk on the day 0, 1, and 3 of induction were 50,000, 900,000, and 1.2 million/mL, respectively, while those on the day 5 and 7 of induction were higher than 2.0 million/mL. In addition, clinical diagnosis showed that the rectal temperature of dairy cows increased significantly after infection, reaching about 40°C. Clinical symptoms were consistent with mastitis, including redness, swelling, pain of mammilla, and fever. Flocculent precipitation was generated in the milk. Besides, HE staining and histopathological identification of the mammary gland tissues on the 7th day after *S. aureus* induction showed that the damaged lobules structure of mammary glands was observed in mammary gland tissues. The acinar cavity was reduced. The breast acinar was partially damaged, and infiltration of inflammatory cells was observed, indicating the onset of severe inflammation of the mammary gland tissues.

### Overview of Sequencing Data

In order to study the expression pattern of miRNA in the blood of dairy cows with *S. aureus-*infected mastitis, peripheral blood sRNA libraries were constructed at different induction stages. The libraries using Illumina Hiseq 2500 platform were sequenced, and raw reads were obtained. High-quality clean reads of more than 18 or <30 nucleotides were retained after removing the untrusted sequences from the sequencing data. The mapped sRNA reads were obtained by comparing clean reads with bovine reference genome using Bowtie software. After quality control, the Q30 of each sample was more than 94.13%, indicating that the sequencing data was reliable and could be used for subsequent analysis (Table 1).

**Table 1:** Overview of miRNA sequencing data.

**Samples**	**dpi**	**Raw reads**	**Q30 (%)**	**Clean reads**	**Mapped small RNA reads**
BS0	0	17,910,689	94.75	16,791,900	10,351,392
BS1	1	21,250,645	94.53	20,120,667	12,446,169
BS3	3	18,142,051	94.42	16,970,040	10,346,316
BS5	5	19,642,620	94.42	17,905,376	11,463,637
BS7	7	21,330,549	94.13	17,327,774	10,530,048

### sRNA Classification

By comparing clean reads with databases (Silva, GtRNAdb, Rfam, and RepBase), miRNA identification was performed, and multiple types of sRNAs were obtained (Table 2). The number of miRNA reads in the peripheral blood of dairy cows at 0, 1, 3, 5, and 7 days of post-infection (dpi) accounted for 84.89, 84.63, 84.77, 83.49, and 77.94% of clean reads, respectively.

**Table 2:** Classification and distribution of small RNAs (sRNAs) at different stages of *S. aureus*-induced mastitis.

**Samples**	**Clean reads**	**rRNA**	**snRNA**	**snoRNA**	**tRNA**	**Repbase**	**miRNA**	**Unannotated** ** small RNA**
BS0	16,791,900 (100.00%)	315,666 (1.88%)	11 (0.00%)	103,825 (0.62%)	55,447 (0.33%)	189,820 (1.13%)	14,254,770 (84.89%)	1,872,361 (11.15%)
BS1	20,120,667 (100.00%)	444,777 (2.21%)	12 (0.00%)	101,641 (0.51%)	41,895 (0.21%)	242,416 (1.20%)	17,028,320 (84.63%)	2,261,606 (11.24%)
BS3	16,970,040 (100.00%)	296,820 (1.75%)	10 (0.00%)	98,715 (0.58%)	21,509 (0.13%)	203,956 (1.20%)	14,385,857 (84.77%)	1,963,173 (11.57%)
BS5	17,905,376 (100.00%)	344,730 (1.93%)	12 (0.00%)	120,251 (0.67%)	100,774 (0.56%)	269,581 (1.51%)	14,948,710 (83.49%)	2,121,318 (11.84%)
BS7	17,327,774 (100.00%)	493,667 (2.85%)	19 (0.00%)	178,604 (1.03%)	168,673 (0.97%)	432,900 (2.50%)	13,505,764 (77.94%)	2,548,147 (14.71%)

### miRNA Identification

**Figure 1:**
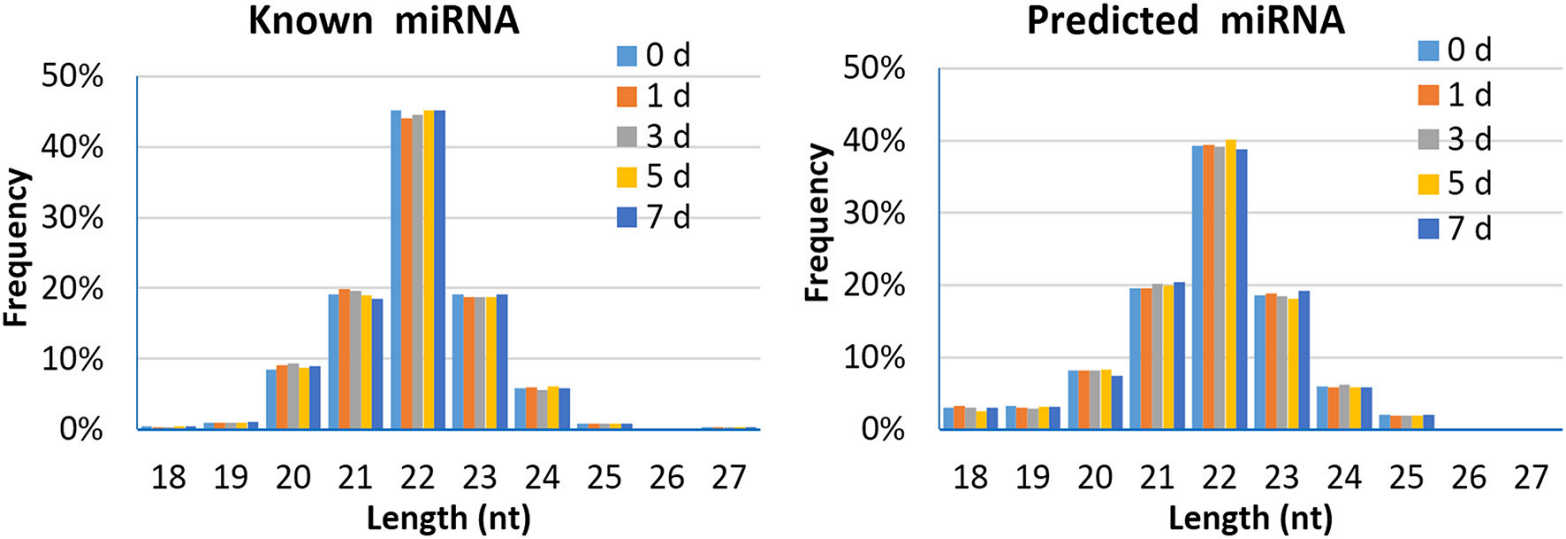
Length distribution of the mapped known miRNA and predicted miRNA in peripheral blood of dairy cows.

**Figure 2:**
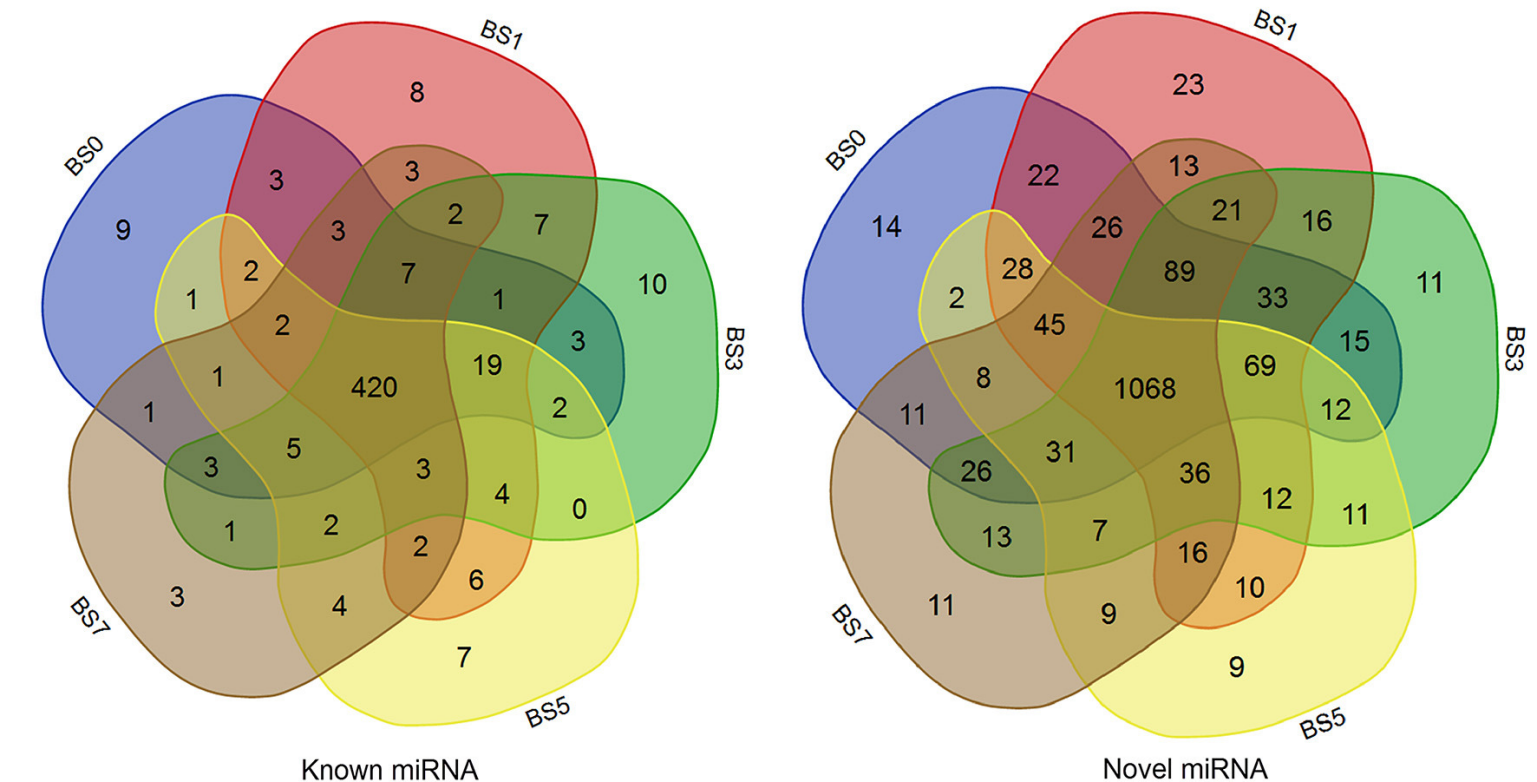
Comparison of miRNA expression in peripheral blood of dairy cows at different times of post-induction (Venn diagram). BS0, BS1, BS3, BS5, and BS7 in the figure represent the groups at 0, 1, 3, 5, and 7 days after *S.aureus* induction.

A total of 2,290 miRNAs were obtained in this study, of which 567 were known miRNAs in miRBase database, and the remaining 1,723 were new miRNAs. The expression quantity of miRNA at each induction time is shown in Table 3. The results of miRNA fragment distribution analysis showed that the main fragment size of known miRNA or the newly predicted miRNA was 22 Nt, followed by 21, 23, 20, and 24 Nt (Figure 1). It was found via comparative analysis of expression quantity of miRNA in five groups that 9, 8, 10, 7, and 3 known DIE-miRNAs and 14, 23, 11, 9, and 11 novel DIE-miRNAs were specifically expressed in the peripheral blood of dairy cows at day 0, 1, 3, 5, and 7 after *S. aureus* induction (Figure 2), indicating that the expression profiles of miRNAs were different at different induction times, which might be related to the onset and progression of mastitis.

**Table 3:** Results of miRNA identification for each sample.

**Samples**	**Known-miRNAs**	**Novel-miRNAs**	**Total**
BS0	498	1,503	2,001
BS1	507	1,533	2,040
BS3	506	1,471	1,977
BS5	496	1,375	1,871
BS7	481	1,434	1,915

### Overall Distribution of miRNA Expression

**Figure 3:**
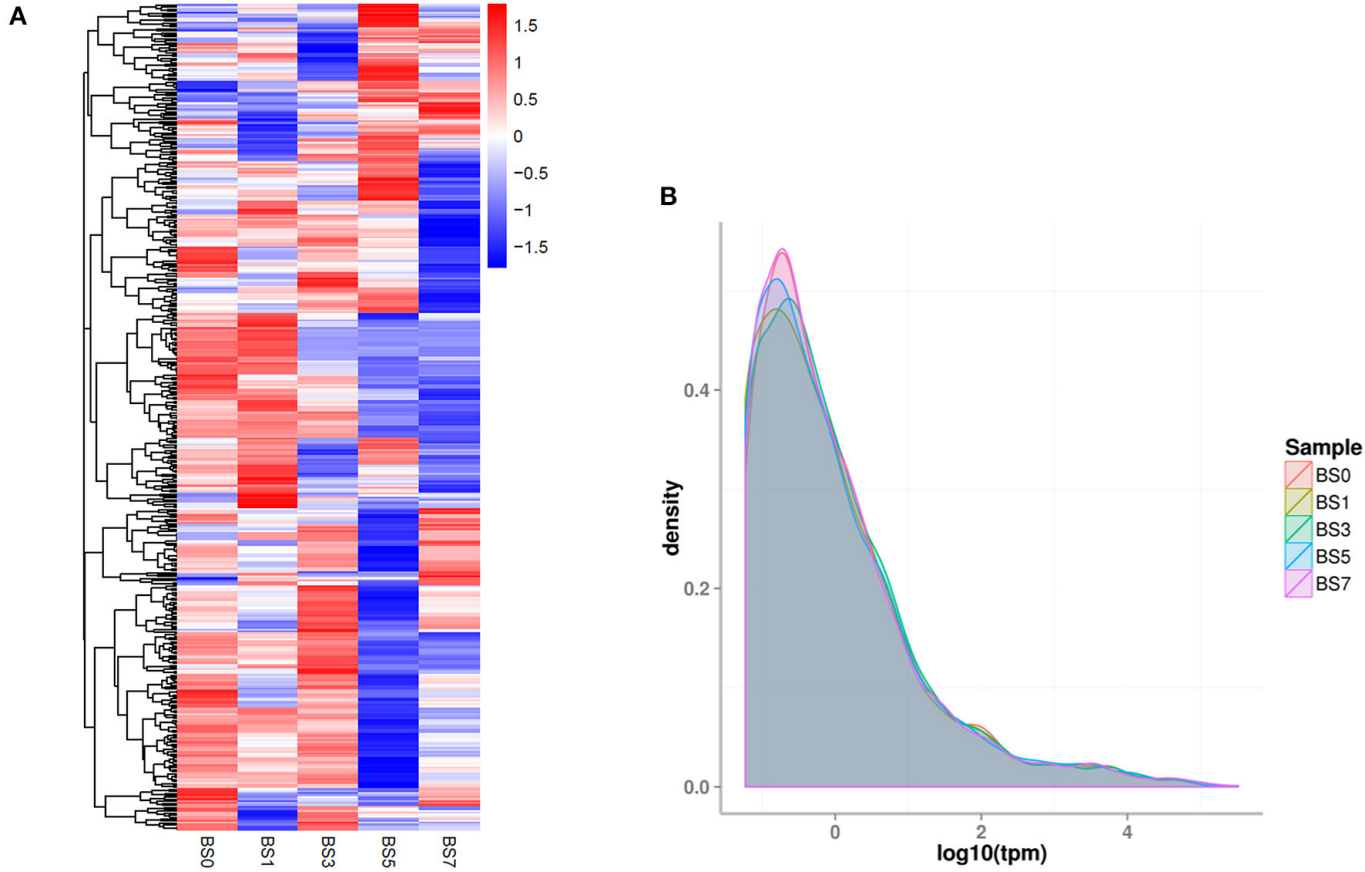
The overall expression profiles of miRNA in each group. **(A)** The heatmap was based on the ${\text{log}}_{2}^{\text{TPM}}$ of each miRNA. **(B)** The density distribution of miRNA expression level (TPM). Different colored curves represent different experimental samples.

Based on clean reads, the expression level of miRNA (TPM) was calculated. The top 50 miRNAs with the highest expression level are shown in Supplementary Table 2. Of these, miR-451 had the highest expression level (TPM value was more than 160,000), followed by miR-486. In addition, a new miRNA unconservative_3_22065 (5′-aaaaaccugaaugacccuuuug-3′) was involved. Compared with the miRNA expression patterns of the five groups, their expression trends were similar, but there were some differences (Figures 3A,B). It was speculated that these DIE-miRNAs might play different regulatory roles in the process of inflammatory response.

### Analysis of miRNA Differential Expression

In this study, a comparative analysis of miRNA expression level differences in blood samples induced by *S. aureus* at different times was conducted. The results showed that 288 DIE-miRNAs were obtained, including 108 known miRNAs and 180 newly predicted miRNAs (Table 4, Supplementary Tables 3–6). Compared with 0 dpi, the number of DIE-miRNAs in 1, 3, 5, and 7 dpi groups were 12, 21, 75, and 48, respectively (Table 4, Supplementary Table 3). With the extension of *S. aureus* infection time, the quantity of DIE-miRNA in blood increased (Table 4), indicating that the response of blood miRNA to inflammation mainly occurred at the late stage of mastitis.

**Table 4:** Number of differentially expressed miRNA in peripheral blood of cow induced by *S. aureus* at different times.

**Comparison**	**Total**	**Up-regulated**	**Down-regulated**
BS0 vs. BS1	12	7	5
BS0 vs. BS3	21	5	16
BS0 vs. BS5	75	19	56
BS0 vs. BS7	48	14	34
BS1 vs. BS3	50	19	31
BS1 vs. BS5	80	18	62
BS1 vs. BS7	80	17	63
BS3 vs. BS5	151	41	110
BS3 vs. BS7	64	16	48
BS5 vs. BS7	90	57	33
Total	288	134	241

**Figure 4:**
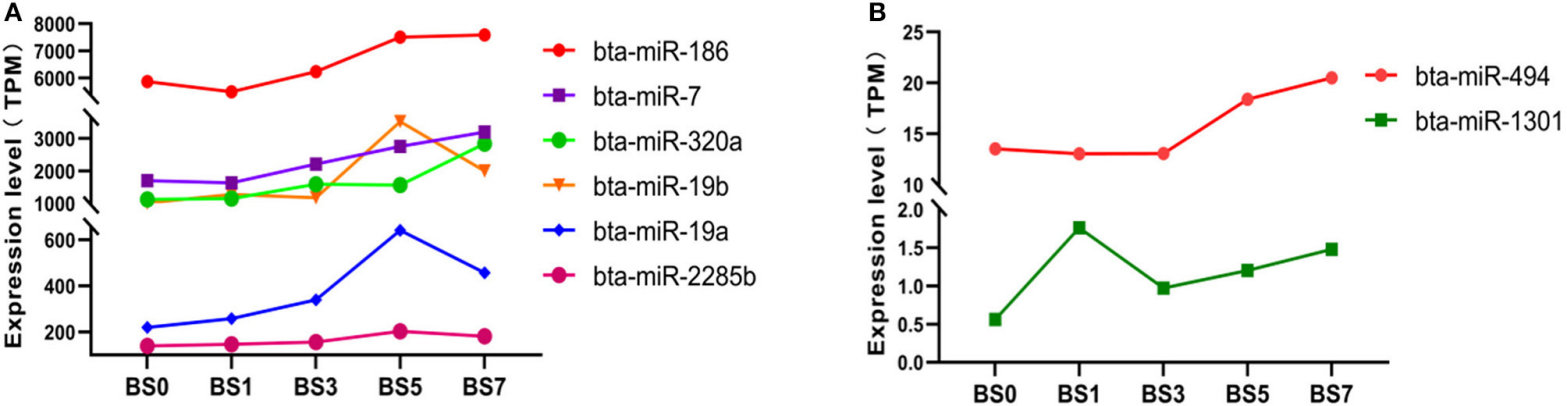
Expression of miRNA with expression partially up-regulated at different induction times. **(A)** miRNA with highly abundant expression; **(B)** miRNA with medium and lowly abundant expression.

The statistical results of expression level showed that the expression of six highly abundant miRNA (miR-186, miR-7, miR-320a, miR-19b, miR-19a, and miR-2285b) (Figure 4A) and two lowly abundant miRNAs (miR-494 and miR-1301) (Figure 4B) increased during *S. aureus* induction. Of these, the expression of miR-320a continuously increased in the blood of dairy cows in the 1, 3, 5, and 7 dpi groups, however, but only in the 7 dpi group did the difference in miR-320a expression reach a significant level (${\text{log}}_{2}^{\text{FC}}$ = 1.3325). miR-19a and miR-19b were significantly up-regulated on the day 5 and 7 after induction (${\text{log}}_{2}^{\text{FC}}$ = 1.5458, 1.0563 and 1.7913, 0.9754, respectively), suggesting that these three miRNAs play an important role in the late stage of cow mastitis. Compared with 0 dpi, the expression of miR-1301 significantly increased on the day 1, 3, 5, and 7 of induction (${\text{log}}_{2}^{\text{FC}}$ was 1.6504, 0.7964, 1.1013, and 1.3998, respectively), which might be used as a blood molecular marker of dairy cow mastitis.

**Figure 5:**
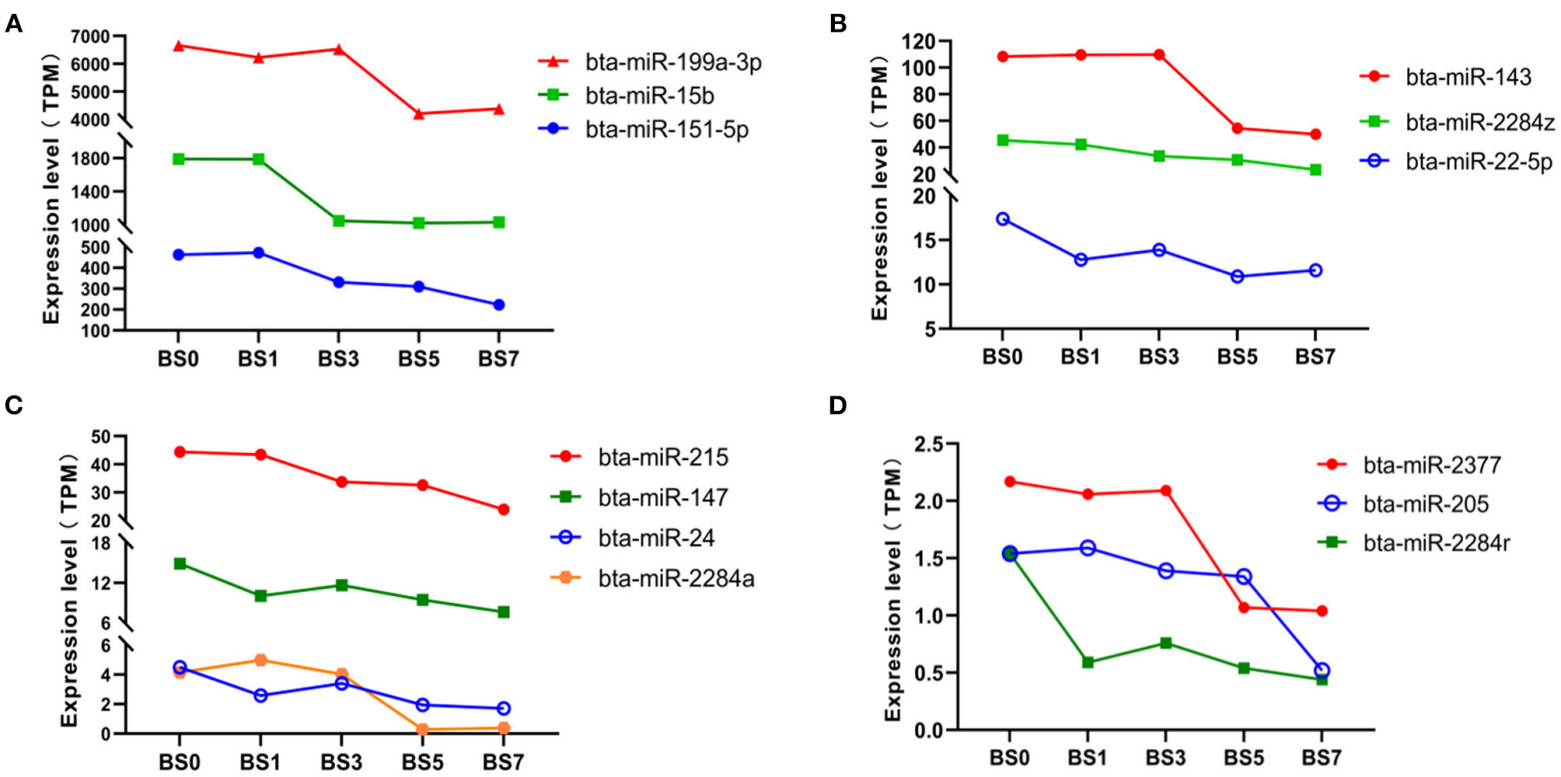
Expression of miRNA with expression partially down-regulated at different induction times. **(A)** miRNA with highly abundant expression; **(B,C)** miRNA with medium abundant expression; and **(D)** miRNA with lowly abundant expression.

We found that there were 13 miRNAs, including 3 highly abundant miRNAs (miR-199a-3p, miR-15b, and miR-151-5p) (Figure 5A), 7 medium abundant miRNAs (miR-143, miR-2284z, miR-22-5p, miR-215, miR-147, miR-24, and miR-2284a) (Figures 5B,C), and 3 lowly abundant miRNAs (miR-2377, miR-205, and miR-2284r) (Figure 5D), showing a decreasing trend of expression at each induction time. Compared with day 0, the expression levels of miR-24 (${\text{log}}_{2}^{\text{FC}}$ = −1.2106 and −1.3986), miR-2284a (${\text{log}}_{2}^{\text{FC}}$ = −3.9512 and −3.4828) and miR-2237 in peripheral blood on the day 5 and 7 significantly decreased (${\text{log}}_{2}^{\text{FC}}$ = −1.0228 and −1.0690), while those of miR-143, miR-151-5p, and miR-205 significantly decreased only on day 7 (${\text{log}}_{2}^{\text{FC}}$ = −1.1137, −1.0550, and −1.5742), suggesting that these miRNAs play an important role in the late stage of *S. aureus*-infected mastitis. The expression levels of miR-215, miR-2284z, and miR-2284r were continuously down-regulated with the prolongation of induction time, and only miR-2284r reached a significant level of difference (${\text{log}}_{2}^{\text{FC}}$ was −1.3940, −1.0132, −1.5280, and −1.7966, respectively), suggesting that miR-2284r can be used as blood molecular markers of dairy cow mastitis.

### qPCR of miR-1301 and miR-2284r in Clinical Samples

**Figure 6:**
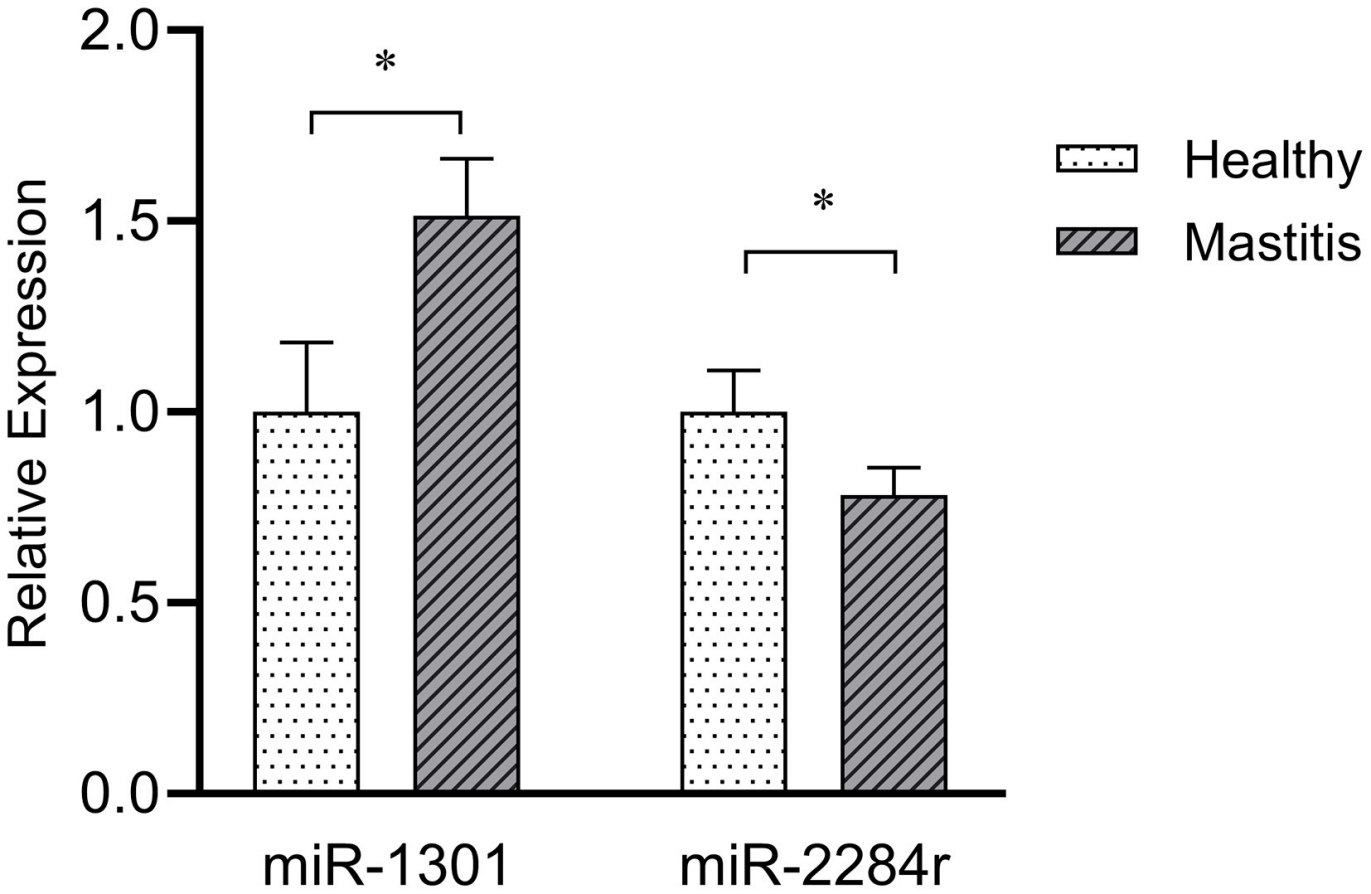
Expression of miR-1301 and miR-2284r in peripheral blood with clinical mastitis comparing to healthy samples. *p < 0.05.

Comparing with the healthy samples, the expression of miR-1301 in clinical samples was significantly up-regulated (*P* < 0.01), while the expression of miR-2284r was significantly down-regulated (*P* < 0.05) (Figure 6), showing a consistent expression trend with the RNAseq results. The above results showed miR-1301 and miR-2284r can be used as a blood molecular markers of cow *S. aureus*-type mastitis.

### Differentially Expressed miRNA qPCR Validation

**Figure 7:**
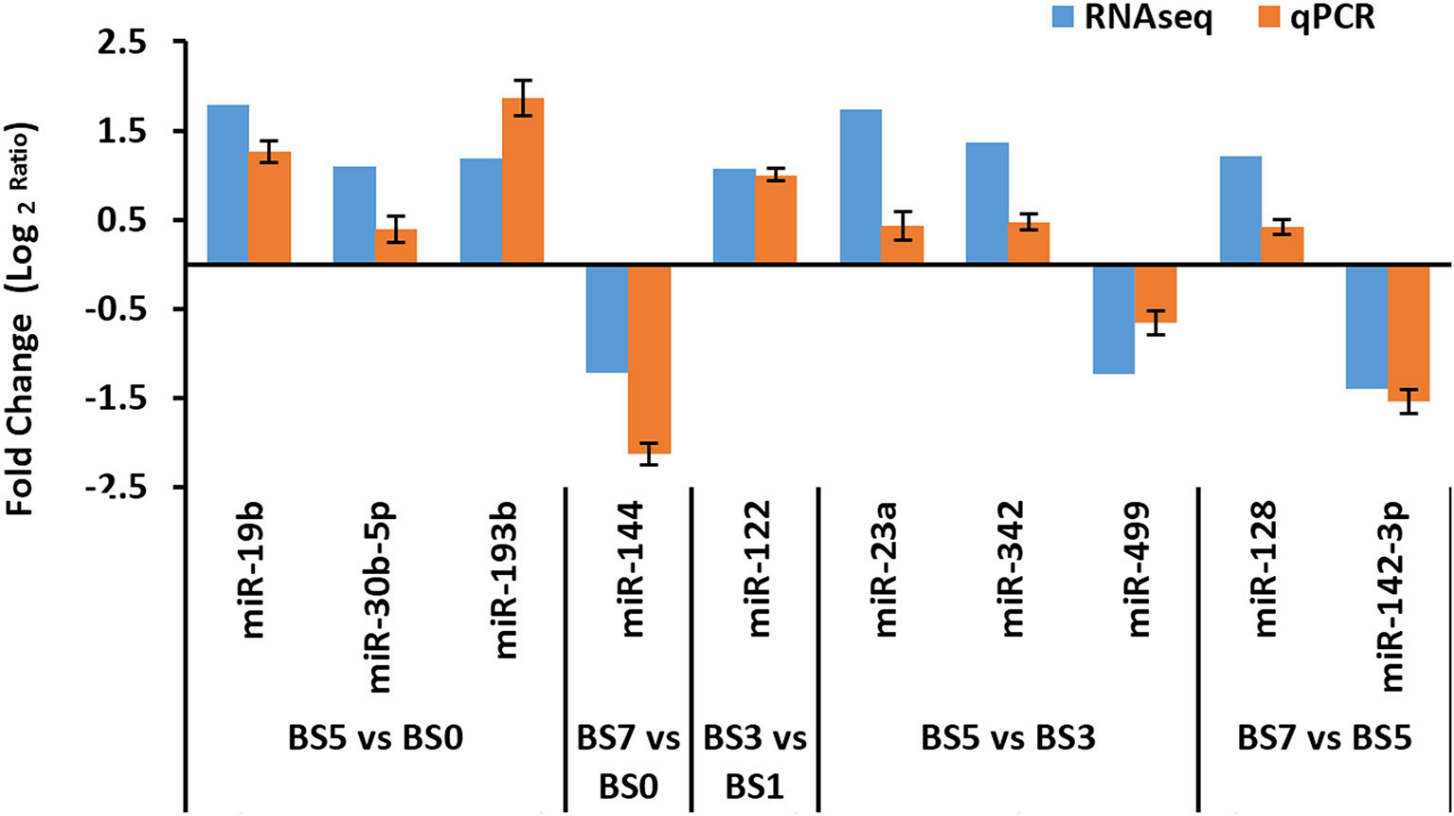
Quantitative polymerase chain reaction (qPCR) verification of differentially expressed microRNA (miRNAs) obtained by RNAseq analysis.

From the obtained DIE-miRNAs, a total of 10 DIE-miRNAs were randomly selected for qPCR quantitative detection. The qPCR results were compared with those of high-throughput sequencing. The qPCR results were completely consistent with those of high-throughput sequencing (Figure 7), indicating that the results of screened DIE-miRNA based on high-throughput sequencing were accurate and reliable.

### Target Gene Prediction and KEGG Function Annotation of DIE-miRNA

**Figure 8:**
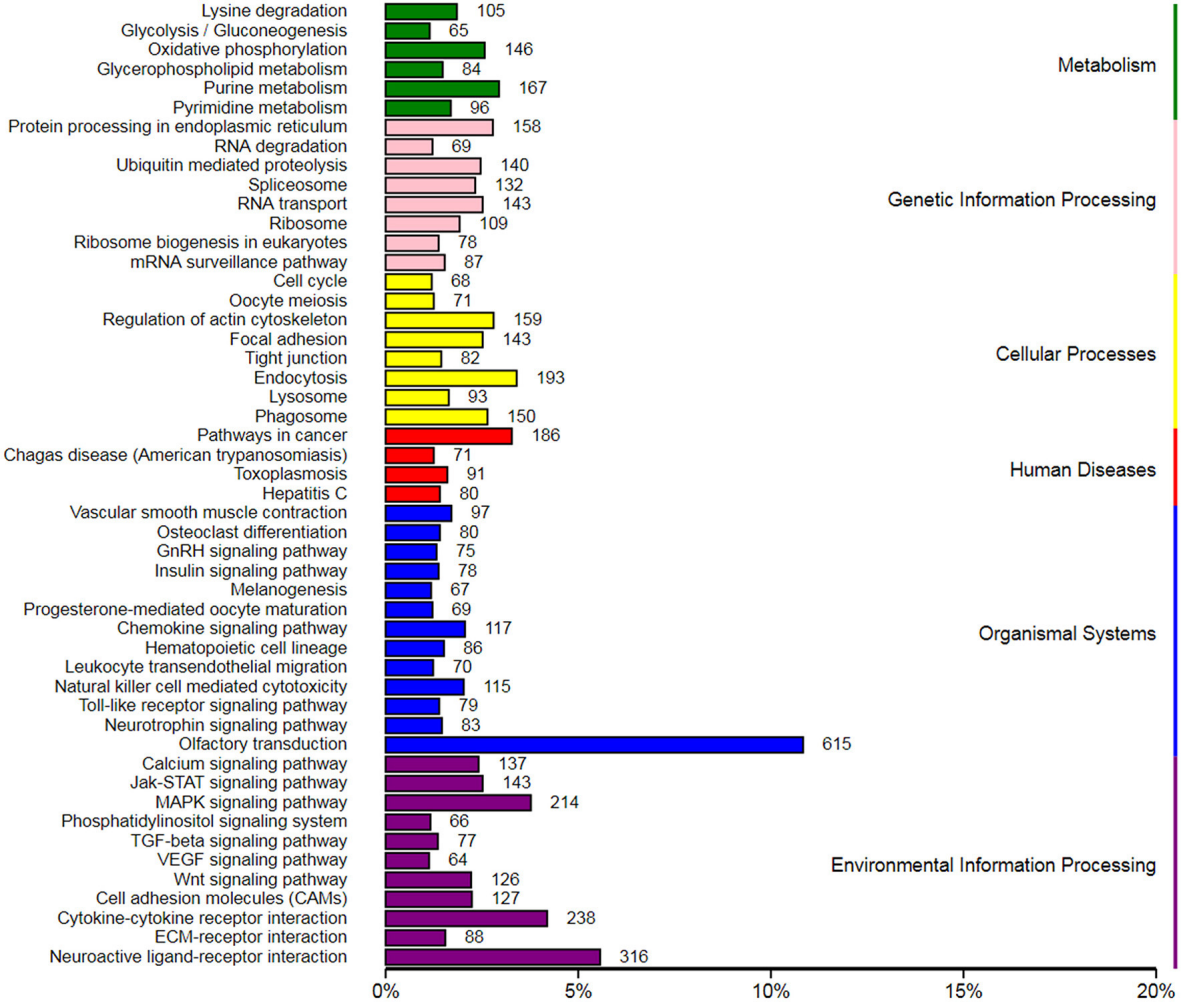
Kyoto Encyclopedia of Genes and Genomes (KEGG) enrichment of target genes of differentially expressed miRNAs in the peripheral blood of dairy cow with *S. aureus*-type mastitis.

In order to explore the role of blood DIE-miRNAs in the progression of *S. aureus*-infected mastitis, a total of 288 DIE-miRNAs might have 18,399 target genes. Their KEGG pathway enrichment analysis indicated that these target genes might be involved in 6 aspects, such as metabolism, genetic information processing, cellular processes, disease, organismal systems, and environmental information processing and 50 distinct signaling pathways. Of these, 10 signaling pathways were related to bacterial infection and immune system, which were chemokine signaling pathway, leukocyte transendothelial migration, natural killer cell-mediated cytotoxicity, toll-like receptor signaling pathway, Jak-STAT signaling pathway MAPK signaling pathway, Wnt signaling pathway, cell adhesion molecules, cytokine-cytokine receptor interaction and ECM-receptor interaction (Figure 8). Therefore, we speculate that these DIE-miRNAs might be involved in regulating the progression of mastitis through the regulation of target genes.

## Discussion

Previous studies have shown that after a person develops a disease, the expression of miRNA in the body changes rapidly to respond and participate in regulating the scope and intensity of the entire disease (21–23). Therefore, the expression level of blood marker miRNA will be changed, which can be used as a biomarker for early diagnosis of diseases and has also been applied to rapid molecular diagnosis of some human diseases (21, 24). Mach et al. (25) reported that blood miRNA could also be used as a molecular marker related to animal production performance. However, there was no report on the blood molecular marker, miRNA of *S. aureus*-infected mastitis. Therefore, it is very necessary to explore the blood molecular markers of *S. aureus* mastitis.

*Staphylococcus aureus* is a common pathogen, which causes mastitis in dairy cows. In order to clarify the molecular mechanism of *S. aureus*-infected mastitis, mastitis animal models (*S. aureus* acute infection for 12 or 24 h) and lipoteichoic acid (LTA) (or inactivated *S. aureus*) induced brain microvascular endothelial cells (bMECs) inflammation model were used to screen differentially expressed genes. Li et al. (8) used Solexa sequencing technology to detect 77 DIE-miRNAs in the mammary gland tissues of dairy cows induced by *S. aureus* for 24 h. Of these, miR-31 and miR-205 were considered to be closely related to breast cancer (26). Fang et al. (27) used high and low doses of *S. aureus* to infect the mammary glands of dairy cows for 24 h. Compared with the control group, DIE-miRNA was detected in both high and low-dose *S. aureus* groups. There was also the differential expression of miRNA between the two infected groups, indicating that the expression level of miRNA was affected by the infection dose of the pathogenic bacteria. In the early stage, we used a low-dose of *S. aureus* to induce the udder of dairy cows for a long time. In the mammary gland tissues after 7 days, a total of 186 miRNA expressions were up-regulated and 93 miRNA expressions were down-regulated. Only a few DIE-RNAs were consistent with other literature reports (8, 9). It was speculated that the induction time or genetic factors of dairy cows would also affect the types and expression levels of differentially expressed miRNAs.

Peripheral blood contains miRNA exosomes, including white blood cells (containing multiple types of genes) and metabolites, which play an important role in immune and disease regulation and can be used to screen some molecular markers for early and rapid diagnosis of diseases. Li et al. (10) detected 123 up-regulated and 55 down-regulated DIE-miRNAs in the peripheral blood of dairy cows with clinical mastitis. However, there was no report on the miRNA expression profiling of peripheral blood of dairy cows with *S. aureus*-infected mastitis. In this study, a total of 288 DIE-miRNAs were detected in the peripheral blood of dairy cows with *S. aureus*-infected mastitis. Bioinformatics analysis showed that they might be involved in regulating the progression of mastitis through cytokine-cytokine receptor interaction, MAPK signaling pathway, Wnt signaling pathway, chemokine signaling pathway, leukocyte transendothelial migration, natural killer cell-mediated cytotoxicity, toll-like receptor signaling pathway, TLR signaling pathways, ECM-receptor interaction and cell adhesion molecules.

According to this study, miR-186 is one of the highly expressed miRNAs in the mammary gland, and its expression increased during the induction process. However, there is no report about miR-186 in human or animal inflammation. Overexpression of miR-186-5p could significantly promote the proliferation of lung adenocarcinoma cells (28). In this study, the expression of miR-186 in the blood of dairy cows after the bacterial infection was increased on the day 3, 5, and 7. Thus, it was speculated that miR-186-5p might be related to cell proliferation and apoptosis in the process of dairy cow mastitis. In addition, the role of miR-186 in diseases may be dependent on its influence on a variety of biological behaviors, including cell proliferation, apoptosis, metastasis, invasion, cell cycle, intracellular metabolism, etc. (29), which needs to be further studied.

miR-320a, miR-19a, and miR-19b were highly expressed miRNAs detected in blood in this study. It was found that the expression levels of these three miRNAs in peripheral blood after *S. aureus* infection were up-regulated, especially the expression levels were all significantly up-regulated at the late stage of *S. aureus* infection (5 and 7 dpi). The current researches on miR-320 have been mostly focused on inflammatory diseases or tumors in humans. Studies have shown that overexpression of miR-320 can down-regulate the expression of AQP1 (30) and SOX4 (31) genes and inhibit epithelial-mesenchymal transition and PI3K/AKT signaling pathway (32) by targeting ELF3 gene, thereby inhibiting the proliferation, metastasis, and invasion of breast cancer cells. In neuronal cells, overexpression of miR-320 affects the proliferation, apoptosis, and oxidative stress damage of ischemic neurons by inhibiting the Nox2/ROS pathway (33). In addition, Matamala et al. (34) showed that the expression of miR-320c was significantly up-regulated in HL60 cells treated with the proinflammatory factor, lipopolysaccharide (LPS), which could be used as a biomarker of the lung inflammation process. miR-19 is an important oncogenic factor of breast cancer, and its expression level is significantly increased in breast cancer tissues and MCF-7 and MDA-MB-231 cell lines. It can reduce cell apoptosis and promote the proliferation of breast cancer cells (e.g., MCF-7) (35–38) by targeting PTEN, tissue factor (TF), interferon (IFN), and MHC I genes (such as HLA-B, HLA-E, HLA-F, and HLA-G). According to the above study, miR-19 plays a key role by regulating genes involved in immune and inflammatory responses and has a potential contribution to cancer cell proliferation, EMT, invasion, metastasis and tumor angiogenesis. The sequences of bovine and human miR-320a, miR-19a, and miR-19b are relatively conservative. Combined with the detection results of expression quantity in this study, it was suggested that these three miRNAs might play an important role in dairy cow mastitis, which might provide potential ideas for analyzing the regulation mechanism of dairy cow mastitis, which should be further studied.

The expression of serum miR-1301-3p was negatively correlated with the pathological staging and metastasis of colorectal cancer, which could be used as a new biomarker for the diagnosis and treatment of colorectal cancer (39). The expression of miR-1301 in HepG2 cells was significantly down-regulated, which could inhibit the metastasis and invasion of HepG2 and cells and promote cell apoptosis, indicating that miR-1301 might be a tumorigenesis inhibitor of HepG2 cells (40). Yang et al. (41) showed that miR-1301 could reduce the Wnt/β-catenin signaling pathway by targeting BCL9, thereby inhibiting the metastasis, invasion and angiogenesis of hepatocellular carcinoma (HCC), which might be a therapeutic target for HCC. So far, there was no report found on miR-1301. Although cancer and inflammation are two different types of diseases, there are many connections between the two. From the perspective of gene regulation level, inflammation and cancer involve common genes or signaling pathways. It can cause metabolic disorders and tissue damage in inflammation, which may trigger cell and tissue pathology and ultimately lead to cancer. This study discovered that the expression of miR-1301 in the peripheral blood at 1, 3, 5, and 7 days of *S. aureus* induction and in clinical mastitis were all significantly up-regulated, suggesting that miR-1301 can be used as a novel blood molecular marker of dairy cow with *S. aureus*-infected mastitis.

The down-regulation of miRNA expression is also related to the onset and progression of diseases. This study found for the first time that the expression of miR-2284a, miR-2237, and miR-24 in the peripheral blood group at 5 and 7 dpi was significantly reduced by more than 2 times, indicating that these 3 miRNAs might have regulatory functions in the late stage of mastitis. There has been no relevant report on miR-2284a and miR-2237 in the inflammatory diseases of other animals (including bovine) and humans. In addition, there was no study reported about bovine miR-24 regulating inflammation. Lin and Yang (42) found that the expression of miR-24 was significantly down-regulated in LPS-induced lung injury in neonatal rats, which was consistent with the results of this study. They also found that miR-24 could reduce the release of proinflammatory factors, TNF-α, IL-6, and IL-1β, and the expression of SP-A and SP-D genes induced by LPS through targeting NLRP3, thereby reducing the inflammatory response of acute lung injury induced by LPS (42). In abdominal aortic aneurysm (AAA) disease, miR-24 is a key regulator of vascular inflammation and AAA pathology, which can be used as a new plasma biomarker of human AAA disease progression (43). The above results indicated that miR-24 could provide a new research direction for the therapy or molecular diagnosis of dairy cow mastitis.

At the same time, this study found that the expression levels of miR-143, miR-151-5p, and miR-205 were significantly reduced at 7 days of *S. aureus* induction. In a previous study, the expression level of miR-205 increased during the pathogenesis in *Escherichia coli-*infected bovine mastitis (11), indicating that the regulatory effects of miRNA might be different in mastitis with varying types of bacterial infection. Kundaktepe et al. (44) reported that the expression level of miR-143 in peripheral blood mononuclear cells of breast cancer patients significantly decreased, which could be used as a breast cancer biomarker. In addition, human miR-143 can inhibit breast cancer cell proliferation and metastasis by regulating the gene expression of MAPK3 (45), MAPK7 (46), MYBL2 (47), CD44 (48), and ERBB1 (49) in cooperation with protein levels and phosphorylation status of AKT, Wnt/β-catenin, SAPK/JNK, and FAK of NF-κB signaling pathways and multiple oncoproteins in JAK/STAT signaling pathway (50). The expression level of miR-143 is related to the worst prognosis, which may be a potential predictor of neoadjuvant therapy response (50). For miR-151-5p, the expression level in the serum of patients with psoriatic arthritis was significantly up-regulated (51). It was speculated that the expression changes of serum miR-151-5p in different inflammatory diseases might be inconsistent, reflecting the functional diversity of miR-151-5. In terms of inflammatory diseases, the expression of miR-205-5b in the serum of LPS-induced sepsis mice (52) was significantly down-regulated, which could inhibit nuclear factor kappa B (NF-κB) signaling, apoptosis and production of proinflammatory factors by targeting high mobility group box protein 1 (HMGB1) and leading to the continuous proliferation of human pulmonary alveolar epithelial cells (53). In addition, miR-205-3p inhibited HCE cell inflammation, oxidative stress, and autophagy by targeting TLR4/NF-κB signaling, thus protecting HCE cells from UV damage (54). Up-regulation of miR-205-5b could be used as a potential therapeutic target for inflammatory diseases, such as and sepsis (52) and lung injury (53). Based on the above-mentioned research results on human or other animals, combined with the above DIE-miRNA dynamic expression results in this study, it was comprehensively inferred that miR-143 and miR-205 might play a regulatory role in dairy cow mastitis, which could provide direction for the biological therapy for *S. aureus* type mastitis.

miR-2284r was newly discovered DIE-miRNA in this study. The expression of miRNA was significantly down-regulated in the blood of dairy cows with clinical mastitis or induction by *S. aureus* at 1, 3, 5, and 7 day. The research on the function of miR-2284r was blank for both humans and other animals. This study has discovered for the first time that miR-2284r might be used as a blood molecular marker for dairy cow mastitis, which still needs further research and verification.

## Conclusion

In this study, 288 differentially expressed miRNAs in peripheral blood of dairy cow with *S. aureus*-infected mastitis were obtained, participating in multiple inflammation-related signaling pathways and reflecting the importance of blood miRNA in inflammation regulation. Furthermore, miR-320a, miR-19a, miR-19b, miR-143, miR-205, and miR-24 might play an important biological role in the late stage of mastitis, providing a direction for the research and development of molecular therapy technology for mastitis. In addition, miR-1301 and miR-2284r can be the novel blood biomarkers for cow *S.aureus*-infected mastitis. The above results laid a new foundation for the research and development of the molecular diagnosis and biological therapy technology for mastitis in dairy cows.

## Data Availability Statement

The original contributions presented in the study are publicly available. This data can be found at: we have uploaded the Raw and processed RNAseq data to the GEO DATABASE (ID GSE172056). The link is https://www.ncbi.nlm.nih.gov/geo/query/acc.cgi?acc=GSE172056.

## Ethics Statement

The animal study was reviewed and approved by Experimental Animal Manage Committee of Northwest A & F University.

## Author Contributions

Z-ML performed the experiments analyzed the data and prepared the draft of the manuscript. X-PW and L-SZ contributed to the conception and design of the study. X-PW, JY, and D-WW contributed to manuscript revision. All authors read and approved the final manuscript.

## Conflict of Interest

The authors declare that the research was conducted in the absence of any commercial or financial relationships that could be construed as a potential conflict of interest.

## Publisher's Note

All claims expressed in this article are solely those of the authors and do not necessarily represent those of their affiliated organizations, or those of the publisher, the editors and the reviewers. Any product that may be evaluated in this article, or claim that may be made by its manufacturer, is not guaranteed or endorsed by the publisher.

## Supplementary Material

Supplementary File 1 RIN of the isolated RNA used for RNAseq.

Supplementary Table 1 The list of primers used for the qPCR validation.

Supplementary Table 2 The expression level (TPM) of the top 50 miRNAs in peripheral blood at different times of mastitis caused by *S. aureus*.

Supplementary Table 3 Differentially expressed miRNAs of peripheral blood with mastitis caused by *S.aureus* with 0 dpi as control group.

Supplementary Table 4 Differentially expressed miRNAs of peripheral blood at different times of mastitis caused by *S. aureus*.

Supplementary Table 5 The list of known DIE-miRNAs indentified by RNAseq.

Supplementary Table 6 The sequences of novel DIE-miRNAs indentified by RNAseq.
