# Supplementary material for: Expression Profiling of microRNA From Peripheral Blood of Dairy Cows in Response to Staphylococcus aureus-Infected Mastitis
Source: Front Vet Sci. 2021 Aug 4;8:691196. doi: 10.3389/fvets.2021.691196 (PMC8371400; doi:10.3389/fvets.2021.691196)
Supplement: Supplementary File 1 — RIN of the isolated RNA used for RNAseq. [file Data_Sheet_1.PDF]

BS0:

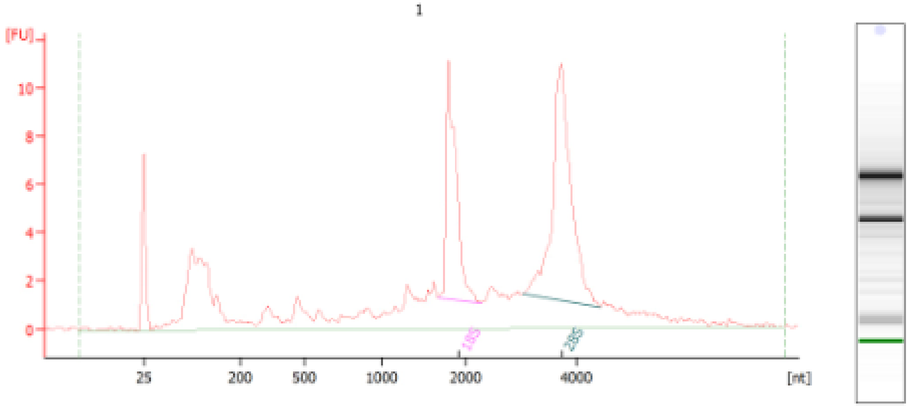

Overall Results for sample 5 : 1

RNA Area:	133.4	RNA Integrity Number (RIN):	8.3 (B.02.08)
RNA Concentration:	93 ng/µl	Result Flagging Color:	
rRNA Ratio [28s / 18s]:	1.5	Result Flagging Label:	RIN: 8.30

Fragment table for sample 5 : 1

Name	Start Size [nt]	End Size [nt]	Area	% of total Area
18S	1,675	2,292	18.2	13.6
28S	3,033	4,412	27.6	20.7

BS1:

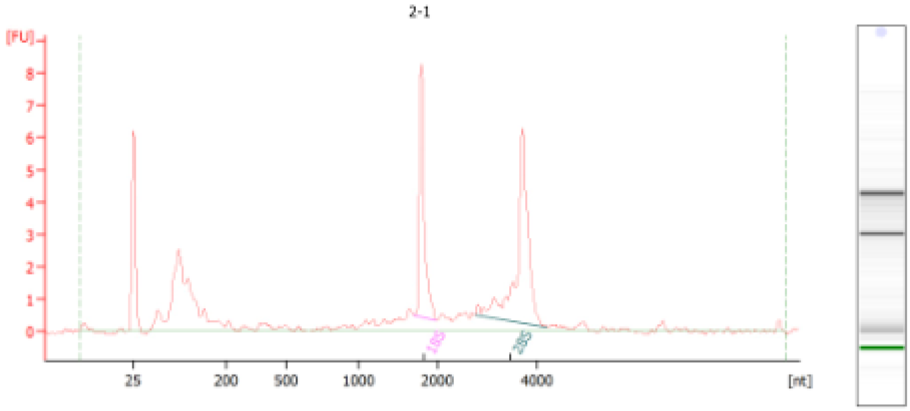

Overall Results for sample 12 : 2-1

RNA Area:	50.3	RNA Integrity Number (RIN):	8.6 (B.02.08)
RNA Concentration:	40 ng/µl	Result Flagging Color:	
rRNA Ratio [28s / 18s]:	1.6	Result Flagging Label:	RIN: 8.60

Fragment table for sample 12 : 2-1

Name	Start Size [nt]	End Size [nt]	Area	% of total Area
18S	1,690	1,979	7.0	13.9
28S	2,758	4,214	11.1	22.1

BS3:

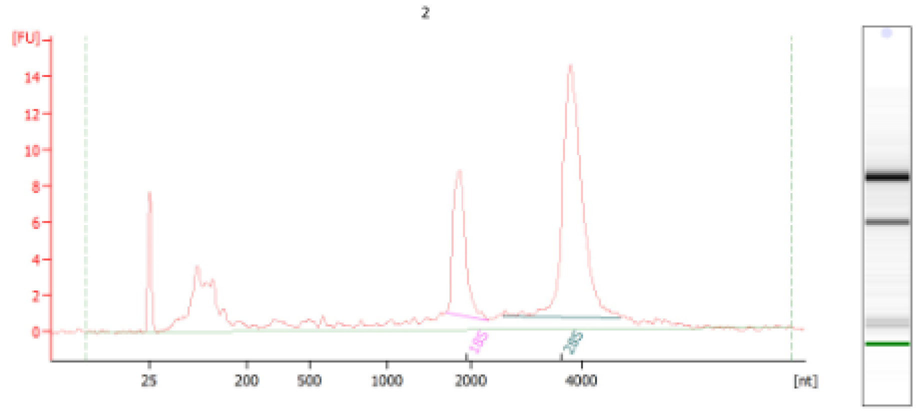

Overall Results for sample 6 : 2

RNA Area:	118.7	RNA Integrity Number (RIN):	9.3 (B.02.08)
RNA Concentration:	83 ng/µl	Result Flagging Color:	
rRNA Ratio [28s / 18s]:	2.2	Result Flagging Label:	RIN: 9.30

Fragment table for sample 6 : 2

Name	Start Size [nt]	End Size [nt]	Area	% of total Area
18S	1,708	2,330	18.2	15.4
28S	2,586	4,698	40.8	34.4

BS5:

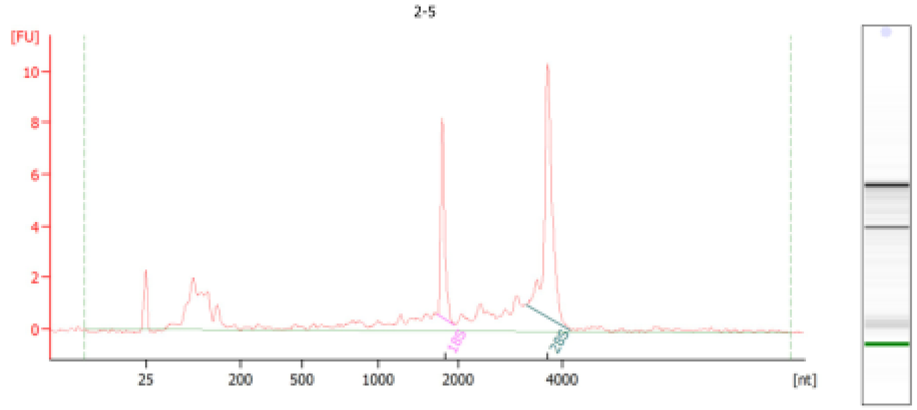

Overall Results for sample 3 : 2-5

RNA Area:	52.1	RNA Integrity Number (RIN):	8.1 (B.02.08)
RNA Concentration:	80 ng/µl	Result Flagging Color:	
rRNA Ratio [28s / 18s]:	2.1	Result Flagging Label:	RIN: 8.10

Fragment table for sample 3 : 2-5

Name	Start Size [nt]	End Size [nt]	Area	% of total Area
18S	1,729	1,941	6.0	11.6
28S	3,296	4,148	12.5	24.1

BS7:

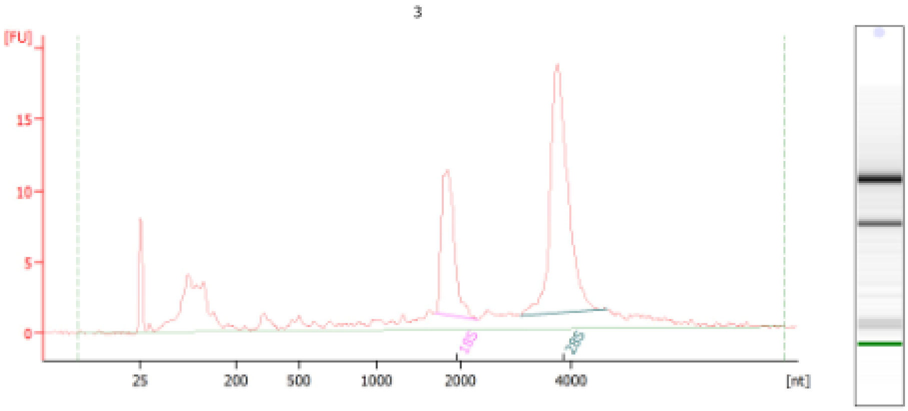

Overall Results for sample 7 : 3

RNA Area:	156.2	RNA Integrity Number (RIN):	9.1 (B.02.08)
RNA Concentration:	109 ng/µl	Result Flagging Color:	
rRNA Ratio [28s / 18s]:	2.0	Result Flagging Label:	RIN: 9.10

Fragment table for sample 7 : 3

Name	Start Size [nt]	End Size [nt]	Area	% of total Area
18S	1,697	2,317	23.8	15.2
28S	3,088	4,669	47.2	30.2
